# Assessment of surface and electrical properties of the TiO_2_@zeolite hybrid materials

**DOI:** 10.1038/s41598-023-30529-8

**Published:** 2023-03-04

**Authors:** G. I. Supelano, F. Mesa, C. A. Parra Vargas, J. A. Mejía Gómez, A. Dussan

**Affiliations:** 1 Grupo Física de Materiales, Universidad Pedagógica y Tecnológica de Colombia (UPTC), Avenida Central del Norte 39-115, 150003 Tunja, Boyacá Colombia; 2 Fundación Universitaria Los Libertadores, Facultad de Ingeniería y Ciencias Básicas, Cra.16 # 63a-68, Bogotá, Colombia; 3 Grupo GIFAM, Universidad Antonio Nariño, Carrera 7 # 21-84, 150001 Tunja, Boyacá Colombia; 4 Grupo de Materiales Nanoestructurados y sus Aplicaciones, Universidad Nacional de Colombia-Bogotá, Cra. 30, No. 45-03, Edificio 404 Lab. 121C, 11001 Bogotá, Colombia

**Keywords:** Photocatalysis, Condensed-matter physics, Materials for energy and catalysis, Structural materials

## Abstract

Degradation of pollutants in aqueous medium is of high interest due to the impact on environment and human health, therefore, design and study of the physico-chemical properties of photocatalysts for water remediation are of major significance. Among properties of photocatalyst, those related to the surface and electrical mechanism are crucial to the photocatalyst´s performance. Here we report the chemical and morphological characteristics of TiO_2_@zeolite photocatalyst by X-ray photoelectron spectroscopy (XPS) and scanning electron microscopy (SEM) respectively, and a coherent electrical conduction mechanism was proposed based on data obtained from assisted laser impedance spectroscopy (ALIS), in which the zeolite was synthesized from recycled coal fly ash. The results obtained by SEM and XPS verified the presence of spherical particles of TiO_2_ anatase with presence of Ti^3+^ state. ALIS results showed that impedance of the entire system increases when the amount of TiO_2_ increases and the samples with lower capacitive performance allowed a larger transfer of the charges between the solid–liquid interface. All results showed that higher photocatalytic performance of TiO_2_ growth over hydroxysodalite with 8.7 wt% and 25 wt% of TiO_2_ can be explained in terms of the morphology of TiO_2_ and the interactions between substrate-TiO_2_ mainly.

## Introduction

Among the dyes, the azo compounds are widely used in the food and textile industries, a considerable amount of the wastewater of those industries are released to the environment which represent a hazard for humans and aquatic life^1,2^. Due to a high chemical stability of dyes the advanced oxidation processes using heterogeneous photocatalysis allows the purification of this class of wastewater^3^. Amid photocatalysts, the TiO_2_ is one of the most used in the photodegradation of pollutants due to its properties such as high oxidation efficiency, chemical and biological inertness, high photostability, ease of production and usage in comparison with other ones, relatively low cost, and environmentally friendly^4,5^; thus, the TiO_2_ has been used and is still studied as a fundamental material in order to improve photocatalyst processes^6–13^. To enhance the performance of TiO_2_, this is grown and dispersed over an adequate substrate creating a hybrid material that uses the properties of both TiO_2_ and substrate. One of the used substrates to immobilize TiO_2_ is the zeolite, which is a hydrated aluminosilicate consisting of TO_4_ (T = Si, Al) tetrahedral units linked through an oxygen atom; they generate a three-dimensional structure with inner cavities and pores of molecular dimensions interconnected by channels. Due to the difference in oxidation state of the Al (+3) and the Si (+4) ions, a negative charge appears, which is neutralized by the exchangeable cations (Na^+^, K^+^, Ca^2+^, Mg^2+^) and by adsorbed water molecules that are placed in the channels or cages of the structure^14,15^. The zeolites are of natural or synthetic origin and are used in numerous applications such as agriculture^16^, health^17^, hydrocarbons^18^, and pollution treatment^19^. Synthetic zeolites can be synthesized from waste materials such as glass and aluminum scraps^20^, coal fly ash (CFA)^21^, lithium waste^22^, and rice husk^23^, among others, by hydrothermal method, mainly. The photocatalyst's ability to degrade pollutants by TiO_2_@zeolite materials is due to the property of the electron–hole pair, promoted by the adsorption of light by TiO_2_ to produce free radicals in an aqueous medium, radicals capable of oxidizing organic compounds as azo dyes^4,5^. The photocatalysis efficiency of the TiO_2_ depends on the number of free radicals produced, which is influenced by the adsorption capability and the recombination of the electron–hole, mainly. The dispersion of TiO_2_ in a zeolite searches to solve any difficulties of the TiO_2_—such as the reduction of the recombination electron–hole rate—, increases the adsorption, and facilitates the recuperation from a liquid solution^24^. According to the literature review, the production of hybrid materials of TiO_2_@zeolite has been reported with commercial zeolite mostly^25–28^; however, the literature related to the use of zeolite synthesized from coal fly ash to the production of TiO_2_@zeolite are scarce^24,29^, consequently a lack of the understanding of the mechanism involved for photocatalysis applications in those materials still.

In a previous work^30^, we reported the synthesis of low cost TiO_2_@zeolite using CFA treated by hydrothermal method. A variation of the experimental parameters allowed to obtain two samples with hydroxysodalite and cancrinite zeolite as a majoritarian phase, these samples were labeled as HYD and CAN respectively. By the impregnation method, different nominal amounts of TiO_2_ (wt%) in the form of titanium isopropoxide were deposited and dispersed on the surface of the HYD and the CAN separately. The structural phase of the TiO_2_ was confirmed to be anatase by employing X-ray diffraction, Raman measurements, and high-resolution transmission electron microscopy. Ti^3+^ state, due to oxygen vacancies in the TiO_2_ structure, was confirmed by electron spin resonance measurements. Photocatalytic degradation of an azo dye was tested with methyl orange under UV radiation; samples with lower amount of TiO_2_ (8.7 wt% and 25 wt%) deposited on HYD showed the higher degradation performance.

In this work we report the study of surface and electrical characteristics of previously synthesized TiO_2_@zeolite hybrid materials; these materials, obtained with concentration different of TiO_2,_ were characterized employing SEM, XPS, and photoimpedance measurements. The study aimed to correlate the structural, morphological, and electrical properties to propose a coherent mechanism about charge transfer that explain the photocatalytic degradation of methyl orange previously reported by this kind of materials.

## Materials and methods

### Materials and synthesis of TiO_2_@zeolite materials

The synthesis of TiO_2_@zeolite is described in ref^30^ briefly. CFA was collected at Sochagota TermoPaipa IV station power plant (Boyacá, Colombia) and passed through 400 sieve mesh (38 μm) with a main chemical composition of Si and Al oxides and Si/Al ratio of 2.4. The CFA was activated by the conventional hydrothermal method. Hydroxysodalite (HYD) zeolite as a majoritarian phase was obtained using 4.2 g of CFA mixed into a distilled water solution of NaOH with a NaOH/CFA ratio of 3.0 at 99.5 °C for 24 h and post-heat treatment at 500 °C. Cancrinite (CAN) zeolite as a majoritarian phase was synthesized using 10 g of CFA mixed into a distilled water solution of NaOH with a NaOH/CFA ratio of 1.7 at 95 °C for 24 h and post-heat treatment at 500 °C.

The synthesis of the hybrid materials was carried out as follows: TiO_2_ was impregnated over two alkaline-activated CFA labeled HYD and CAN. Different nominal amounts of TiO_2_ (wt%), in the form of titanium isopropoxide, were added drop by drop to a solution of ethanol with HYD and CAN, separately, for six hours, under magnetic stirring, at 60 °C. Solutions were dried overnight at 90 °C in a muffle furnace. The hybrid materials were labeled relative to the scheme of Table 1.

**Table 1:** Labeling of samples of the hybrid materials.

TiO_2_ impregnated over HYD	TiO_2_ impregnated over CAN	TiO_2_ (wt%)
HYD-T-8.7	CAN-T-8.7	8.70
HYD-T -25	CAN-T-25	25.00
HYD-T -33.25	CAN-T-33.25	33.25
HYD-T -41.3	CAN-T-41.3	41.30
HYD-T -49.45	CAN-T-49.45	49.45

### Characterization

The surface chemical analysis was carried out by X-ray photoelectron spectroscopy, XPS, using an X(NAP-XPS) spectrometer with a PHOIBOS 150 1D-DLD analyzer, employing an excitation source of Al-Kα (1486.7 eV, 13 kV, 100 W) with a step of 20 eV and 0.1 eV. All the spectra were calibrated with a C1s electron peak at 284.6 eV. The morphological surface features were observed by scanning electron microscopy (SEM) using a piece of LYRA3 TESCAN equipment in backscattering and secondary modes, and the chemical composition was obtained by Energy Disperse X-ray Spectroscopy (EDS).

The electrical properties were studied employing assisted laser impedance spectroscopy (ALIS) measurements in darkness and under illumination, using an unpolarized TEM 00 solid-state laser of wavelength λ = 390 nm, with a power of 35 mW/cm^2^. The data were collected using a homemade Bayonet Neill–Concelman connector (BNC)-based breakout box to a Keysight E4980AL-precision LCR meter. The measurements were achieved with an AC signal of 100 mV amplitude in a frequency range between 20 Hz and 1 MHz and with zero DC bias. Data were fitting using two equivalent electrical circuits using the EIS Spectrum Analyser 1.0 software^31^. The sample was placed in a cell of cylindrical shape comprised of two isolated stainless-steel electrodes separated by a polytetrafluoroethylene O-ring. 50 mg of the hybrid materials were diluted in 200 mL of MilliQ water; from this solution, a volume of 200 mL was used to make the measurement.

## Results and discussion

**Figure 1:**
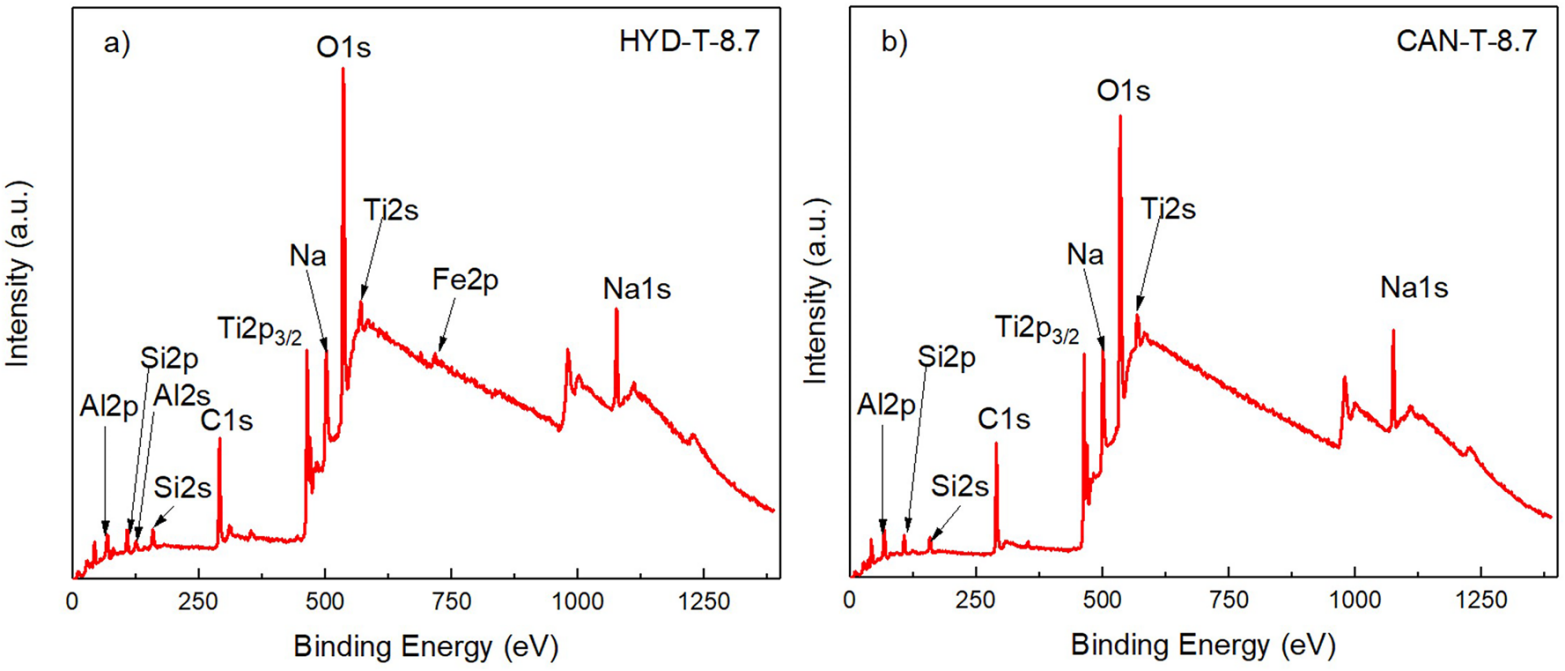
Wide XPS spectra of the samples (**a**) HYD-T-8.7 and (**b**) CAN-T-8.7.

XPS spectra measurements were obtained to analyze the electronic and surface chemical states of the samples. Figure 1a,b shows the full XPS spectrum of the HYD-T-8.7 and CAN-T-8.7 samples, respectively. The spectrums show signals from Al, Si, Na, and Ti. Additionally, CAN-T-8.7 exhibits a signal originating from Fe. Al, Si, and Na arise from the zeolites and other aluminosilicates rising from alkaline-treated CFA; the Ti emerges from the impregnated TiO_2_ mainly, and the Fe emerges from the CFA. All hybrid materials, CAN-Ts and HYD-Ts, display similar spectrums.

**Figure 2:**
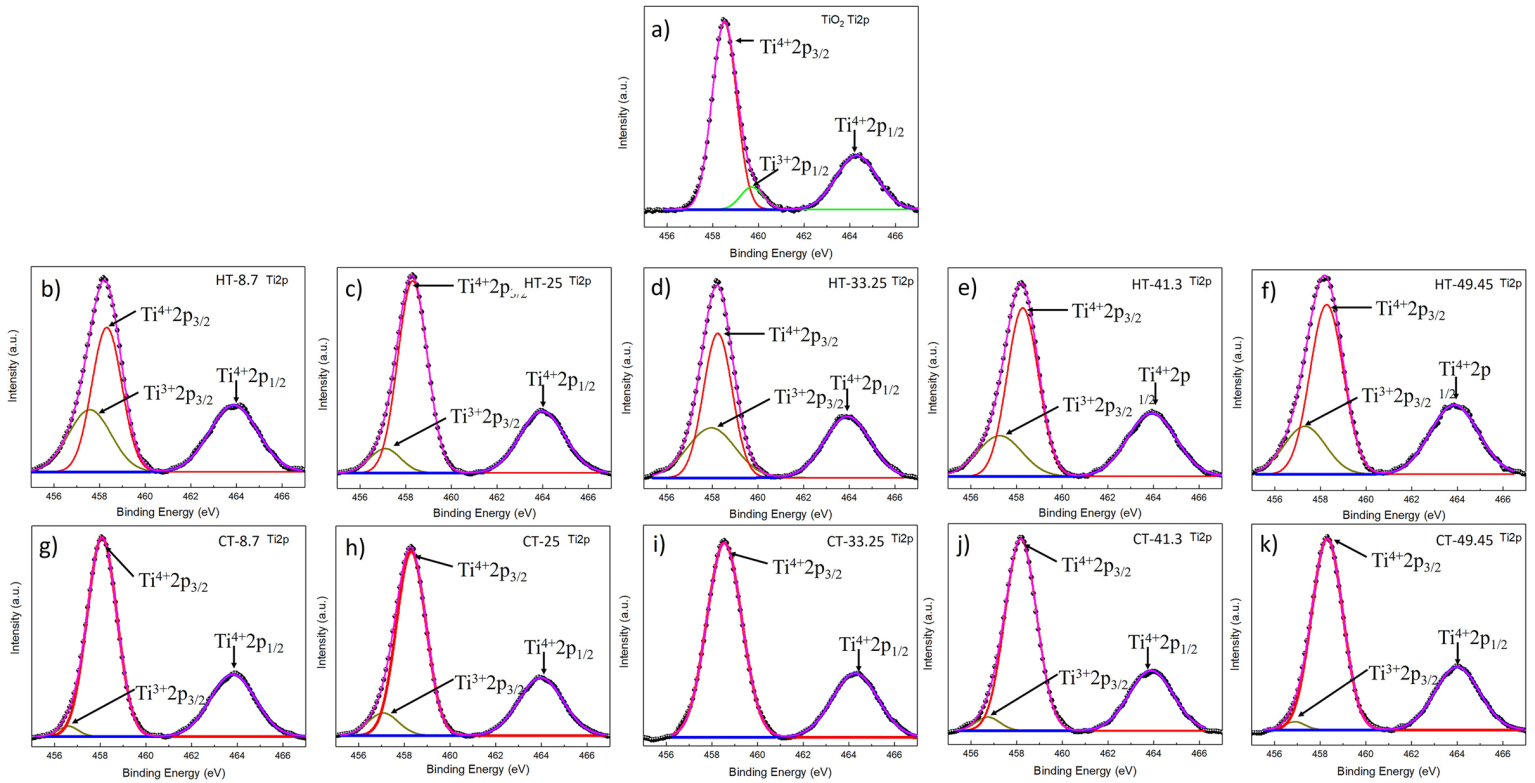
Deconvoluted spectra of the Ti2p for, (**a**) synthesized TiO_2_ (**b–f**) HYD-T and (**g–k**) CAN-T hybrid materials.

**Figure 3:**
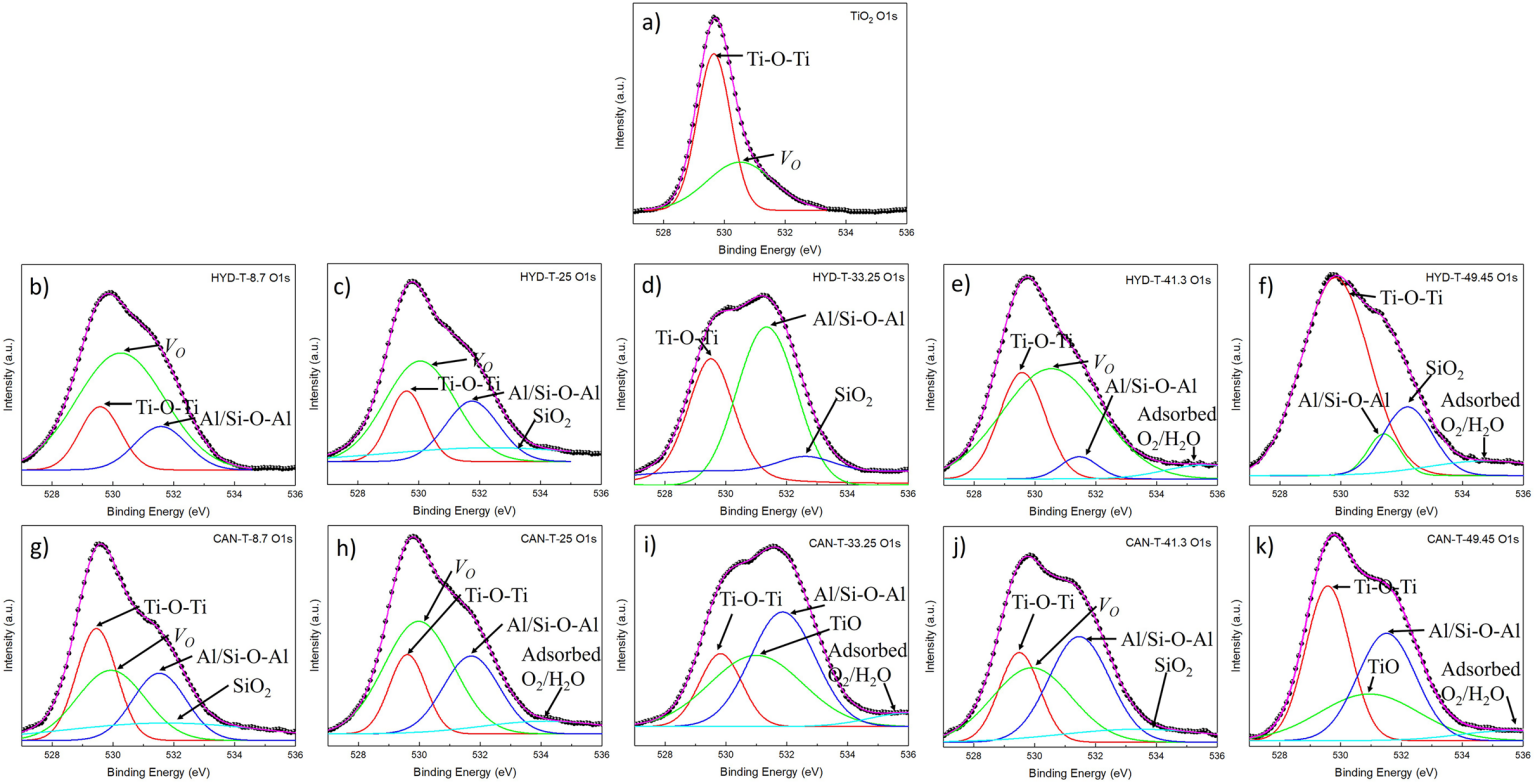
Deconvoluted spectra of the O1s for (**a**) synthesized TiO_2_ (**b–f**) HYD-T and (**g–k**) CAN-T hybrid materials.

High resolution XPS spectra was performed to analyze the peaks corresponding to Ti2p (Fig. 2) and O1s (Fig. 3). These spectra were fitted with a Gaussian-peak function.

For synthesized TiO_2,_ two peaks at 458.5 eV and 464.4 eV emerge from spin–orbit splitting. The deconvoluted spectra for synthesized TiO_2_ contains main peaks at 458.5 eV, 459.7 eV, and 464.3 eV that can be assigned to Ti^4+^2p3/2^12^, Ti^3+^2p1/2^13^, and Ti^4+^2p1/2^32,33^ respectively, Fig. 2a. The peaks corresponding to Ti^4+^ state is attributed to the Ti^4+^ of the TiO_2_ lattice; the peak corresponding to Ti^3+^ state is attributed to oxygen vacancies in the surface.

In all samples, HYD-T (Fig. 2b–f) and CAN-T (Fig. 2g–k) the peaks appear at around 458.5 eV and 464.3 eV, both corresponding to the Ti^4+^ state. The peak at around 459.7 eV of the Ti^3+^ state does not arise ; however, one third signal emerges at around 456 eV, which corresponds to the Ti^3+^2p3/2 state^32^. For CAN-T-33.25, the sample contribution of the Ti^3+^ state was not observed (Fig. 3i).

For synthesized TiO_2,_ the O1s XPS high-resolution spectra contain main peaks at 529.7 eV and 530.5 eV that can be attributed to Ti–O–Ti^34^ and an oxygen vacancy (VO)^33^, respectively (Fig. 3a).

The O1s XPS spectra for both HYD-T (Fig. 3b–f) and CAN-T (Fig. 3g–k hybrid materials depict extra peaks; these were deconvoluted with peaks at 529.7 eV, 530.5 eV, 531 eV, 531.5 eV, 532.4 eV, and 534.8 eV, which can be attributed to Ti–O–Ti^34^, oxygen vacancy^33^, Ti–O^12^, Al–O–Al/Si–O–Al^35^, SiO_2_^35^, and adsorbed O_2_/H_2_O^34^, respectively.

**Figure 4:**
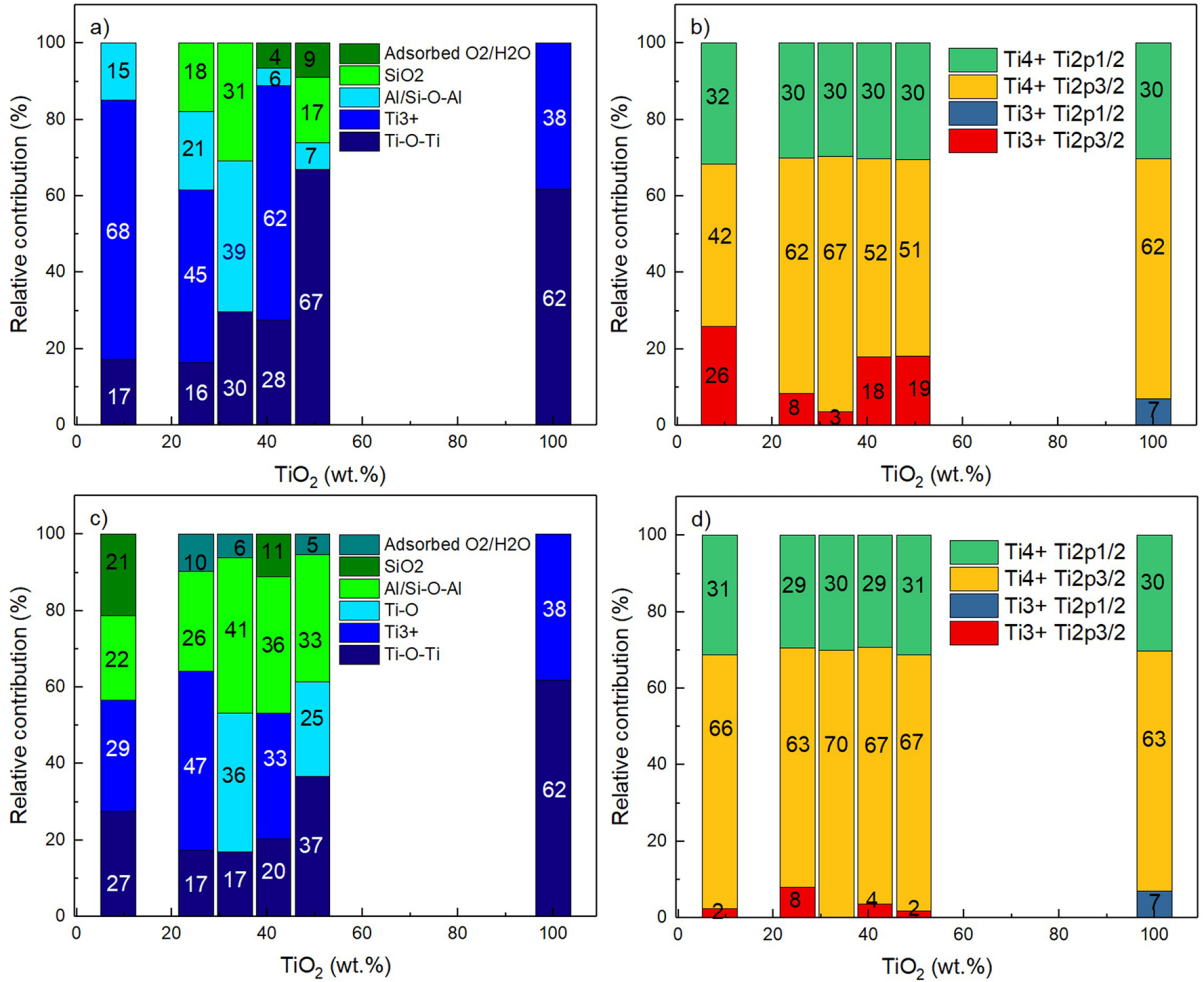
Relative contribution to the (**a**) O1s, (**b**) Ti2p peak for HYD-T; (**c**) O1s and (**d**) Ti2p peak for CAN-T hybrid materials.

The relative contribution of the deconvoluted signal to the total spectra of Fig. 2. and Fig. 3. for each of the samples is provided in Fig. 4. The bars at 100 wt% of TiO_2_correspond to the contribution of the synthesized TiO_2_. The high percentage of titanium in the hybrid materials refers to a high content of TiO_2_on the substrate surface. For the HYD-T samples, the Ti^3+^ contribution decreases when the TiO_2_load increases until 33.25 wt% TiO_2_, then the Ti^3+^ contribution increases when TiO_2_load increases. Concerning the CAN-T samples, the Ti^3+^ contribution increases when the TiO_2_load increases until 25 wt% TiO_2_, then the Ti^3+^ contribution is zero for 33.25 wt% TiO_2_, next, the Ti^3+^ contribution arise for 41.3 wt% TiO_2_, this contribution is smaller than the contribution for 25 wt% TiO_2_and finally, the Ti^3+^ contribution decreases for 49.45 wt% TiO_2_. The results show that the contribution of the Ti^3+^ state in the TiO_2_phase on the surface of HYD-T samples behaves contrary to CAN-T samples as a function of the amount of loaded TiO_2_.

**Figure 5:**
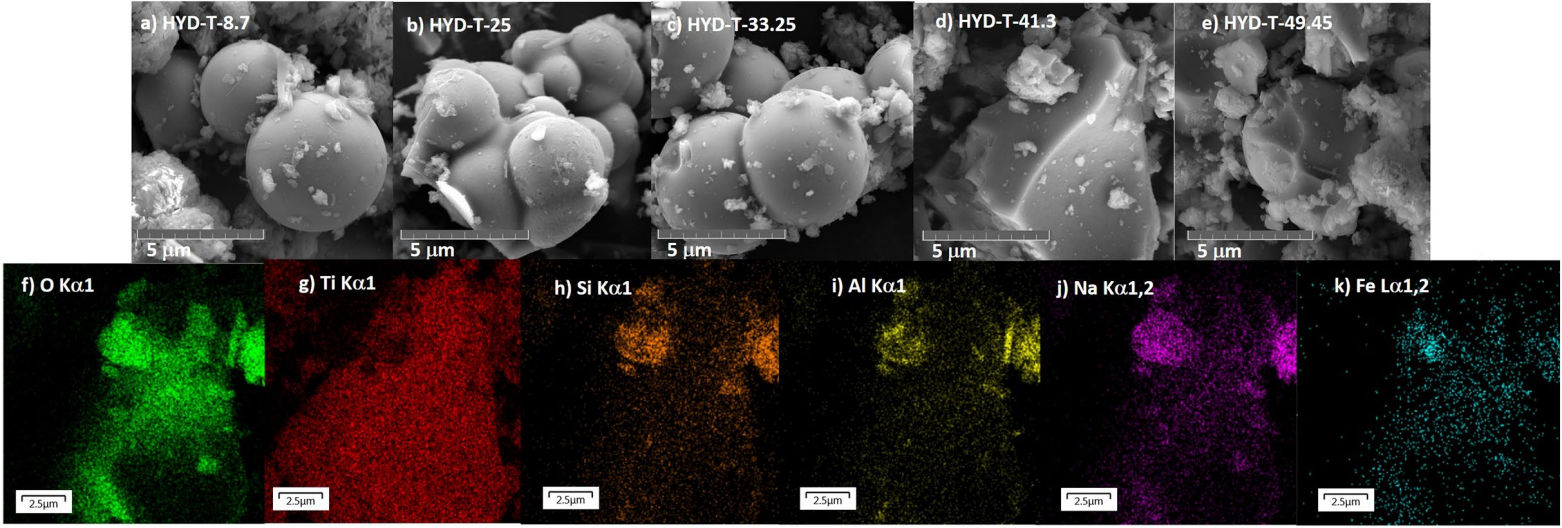
SEM images of the hybrid materials HYD-T for (**a**) 8.7, (**b**) 25, (**c**) 33.25, (**d**) 41.3 and, (**e**) 49.45 wt% TiO_2_, and (**f–k**) EDS map of the sample HYD-T-41.3 related to the Fig. 2d.

Figure 5. displays the SEM images of HYD-T materials. It can be depicted that the morphology of the materials is composed of particles with two distinctive shapes, mainly. Particles are conspicuously spherically shaped in HYD-T-8.7, HYD-T-25, and HYD-T-33.25. The morphology of TiO_2_ changes for higher TiO_2_ amounts in which the particles have truncated faces in HYD-T41.3 and HYD-T49.45 samples, both shapes with smooth surfaces. These particles correspond to TiO_2_. It is noticed that aggregation takes place in TiO_2_ particles (it is evident in HYD-T-25), and it makes that particle size bigger than 2.5 mm. Particles with rod and flake shapes correspond to aluminosilicates, and the grade of aggregation is smaller than that in TiO_2_ particles. The aggregation of TiO_2_ particles indicates that the TiO_2_ intraparticle attachment rate is larger than the TiO_2_-substrate attachment rate. Elemental composition was obtained through an EDS probe. Figure 5f–k shows the EDS map for HYD-T-41.3.

**Figure 6:**
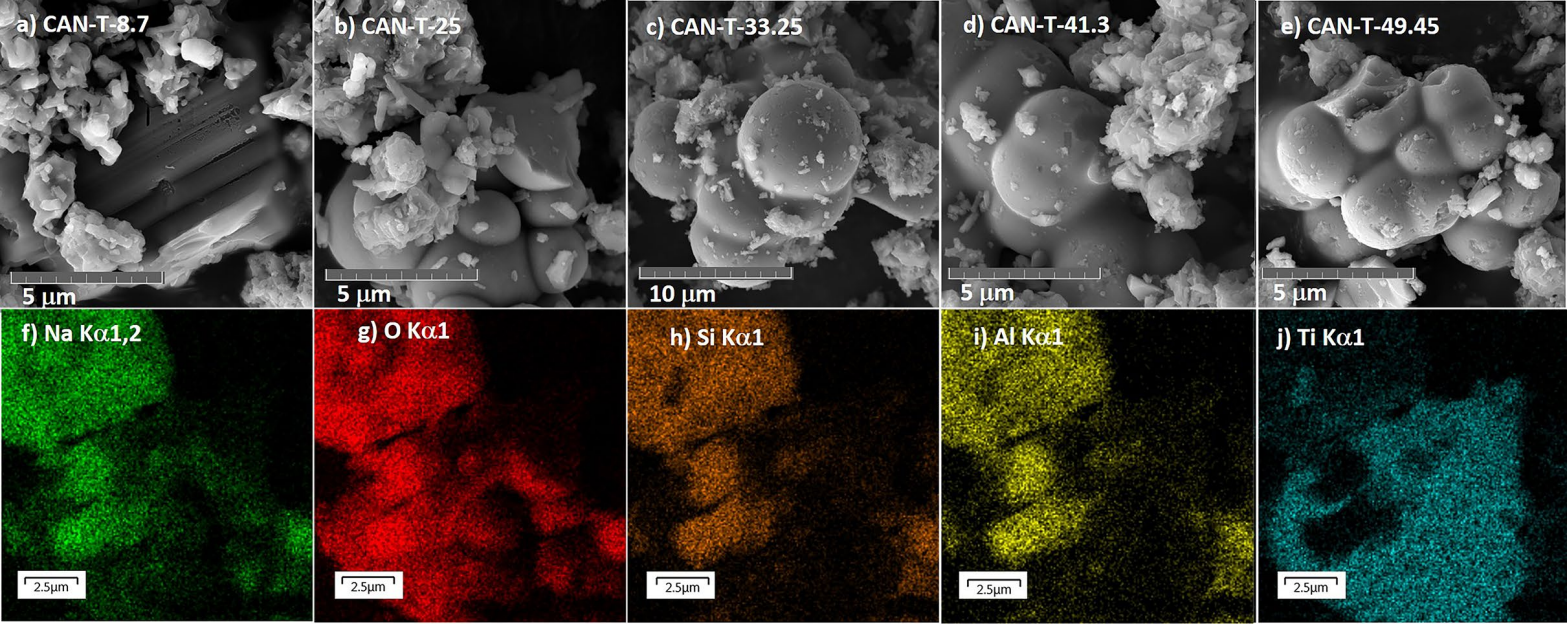
SEM images of the hybrid materials HYD-T for (**a**) 8.7, (**b**) 25, (**c**) 33.25, (**d**) 41.3 and, (**e**) 49.45 wt % TiO_2_, and (**f**) EDS map of the sample CAN-T-25 related to the Fig. 4b.

Figure 6 displays the SEM images of CAN-T materials. It can be seen that the morphology of the materials exhibits similar behavior to that of the HYD-T samples. The spherical shape is predominant in TiO_2_ particles for CAN-T-25, CAN-T-33.25, CAN-T-41.3, and CAN-T-49.45; for smaller amounts of TiO_2_, the morphology changes to truncated faces in CAN-T-8.7. The surface of the TiO_2_ particles exhibits a smooth surface. The aggregation takes place in TiO_2_ particles, and it is larger in comparison with the aggregation of TiO_2_ particles in HYD-T samples. Particles with bar and flake shapes correspond to aluminosilicates. The TiO_2_-substrate attachment in this sample is larger in comparison with that depicted in HYD-T samples. Figure 6e–j shows the EDS map for HYD-T-25. The results show that elemental composition is due to the substrate’s aluminosilicates and TiO_2_, mainly with the presence of other elements, such as iron, which come from treated CFA.

**Figure 7:**
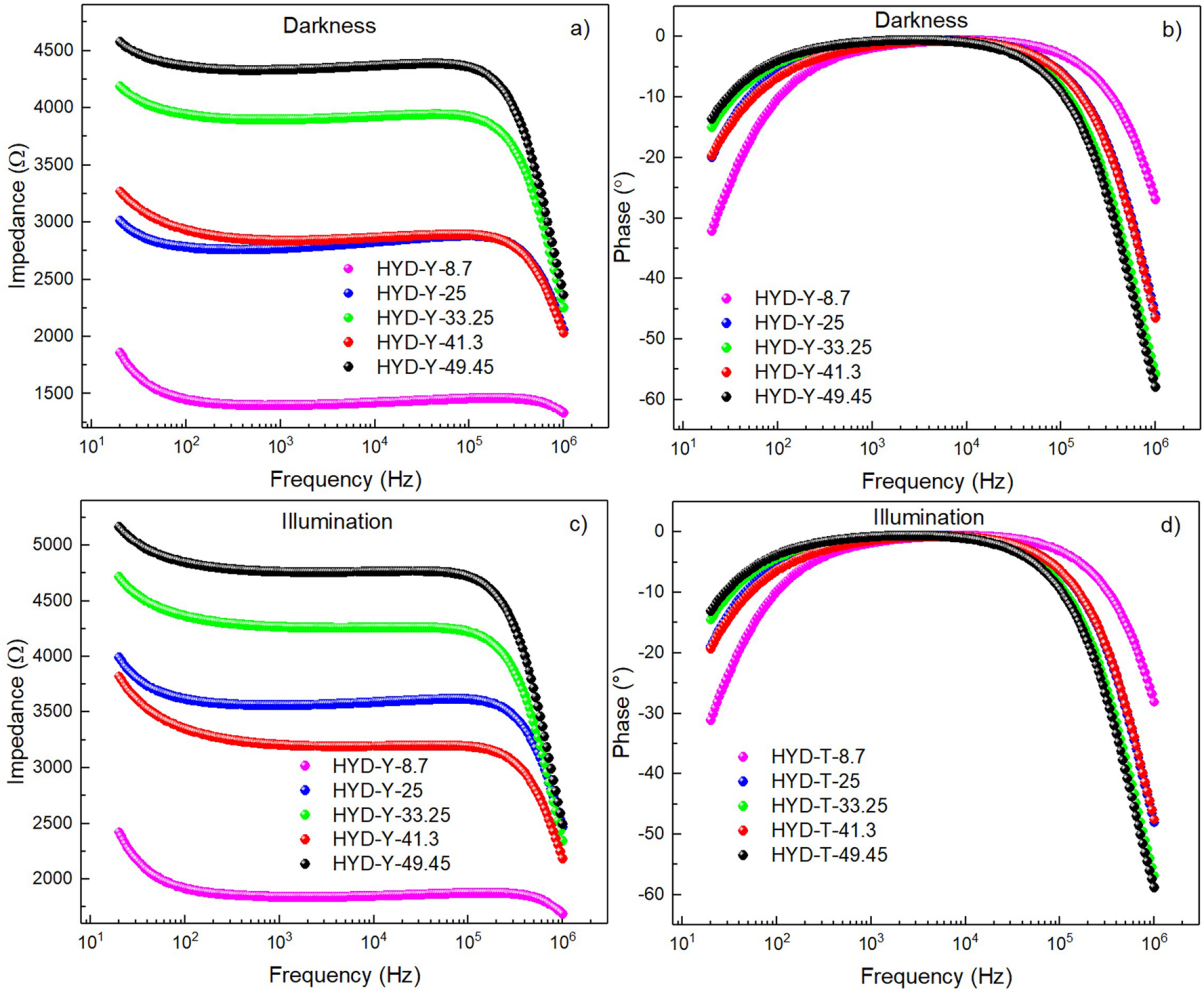
Bode plots of the samples HYD-T in dark (**a**) impedance, (**b**) phase; under illumination, (**c**) impedance, and (**d**) phase.

**Figure 8:**
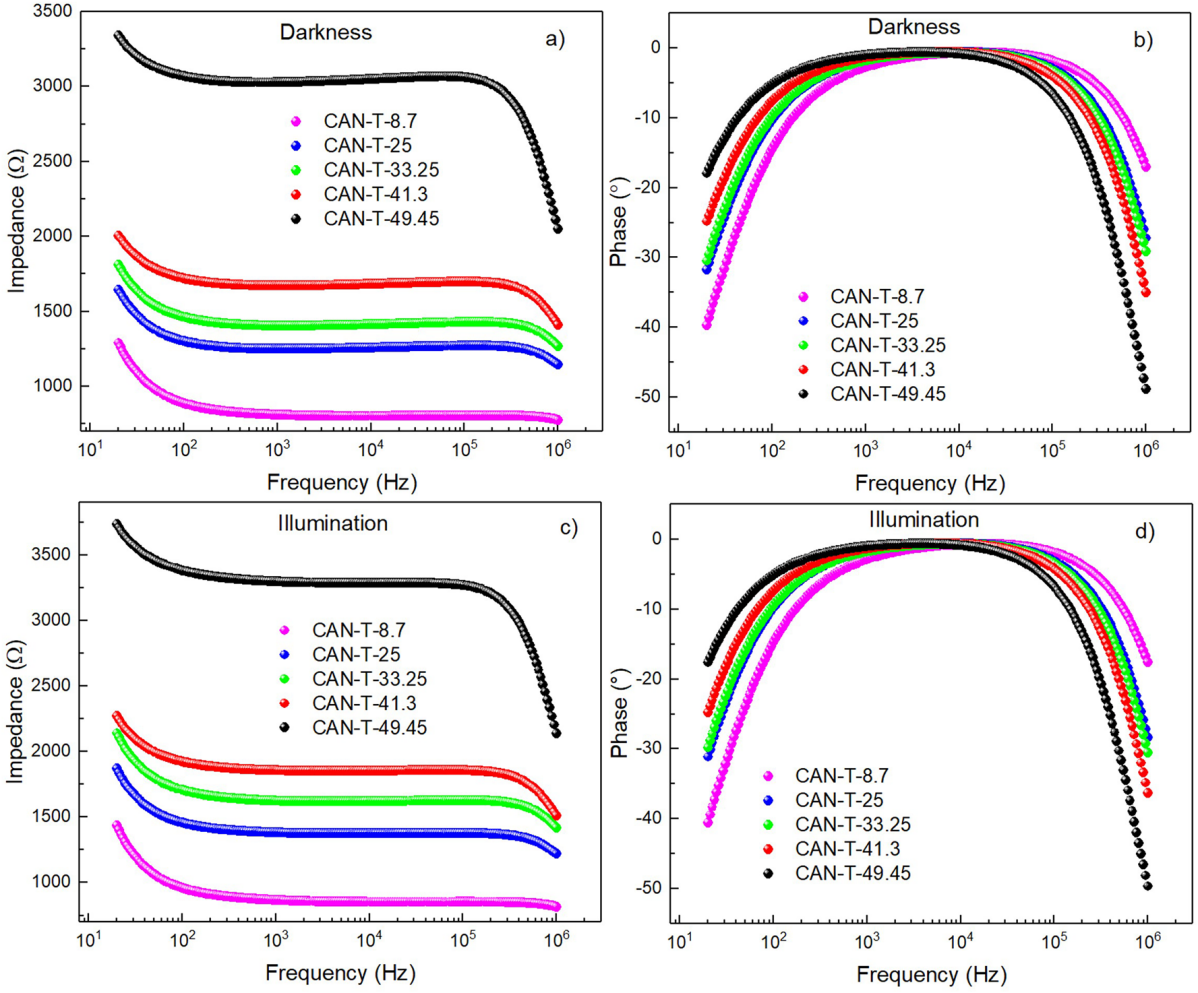
Bode plots of the samples CAN-T in dark (**a**) impedance, (**b**) phase; under illumination, (**c**) impedance, and (**d**) phase.

In order to investigate the electrical behavior of the samples, ALIS response were measured in darkness and under UV light, from these measurements the Bode plots of the HYD-T and CAN-T were obtained and are shown in Figs. 7 and 8 respectively. The data reveal an increase of the impedance when the sample is illuminated with a resistive behavior at frequencies between 10^3^ and 10^5^ Hz, and a capacitive behavior below 10^3^ Hz and above 10^5^ Hz. The impedance tends to increase when the amount of TiO_2_ increases.

**Figure 9:**
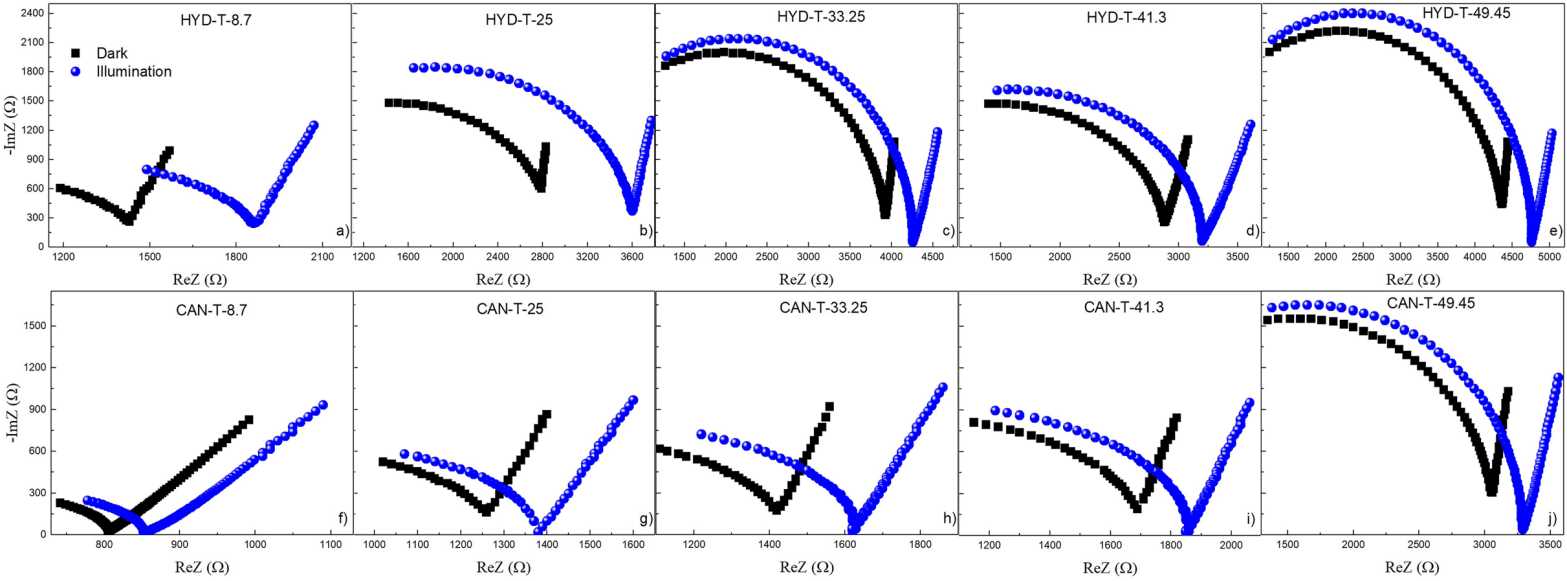
Nyquist plots of the samples HYD-T and CAN-T in darkness and under illumination.

Figure 9 displays the Nyquist plots in darkness and under illumination for both series of materials, HYD-T and CAN-T. The curves exhibit two semicircles. The curvature radii of the semicircle at low frequency is larger than that at high frequency, which increases when the quantity of TiO_2_ increases. The curvature radii in each sample increase when radiated.

**Figure 10:**
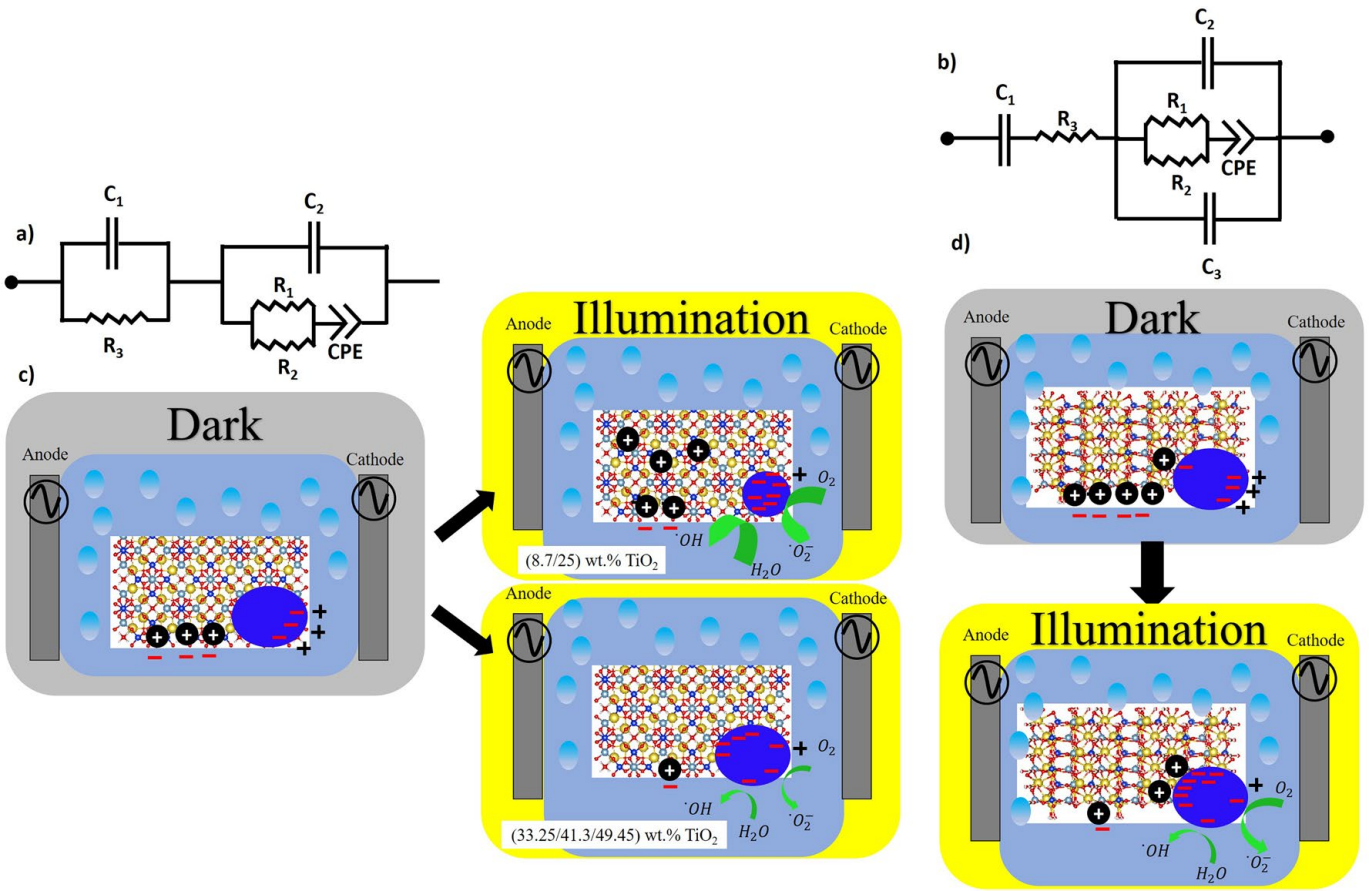
Equivalent circuit for (**a**) HYD-T and (**b**) CAN-T hybrid materials; schematic processes in the (**c**) HYD-T and (**d**) CAN-T hybrid materials.

Nyquist plots of both HYD-T and CAN-T materials in darkness and lighting conditions were fitted to an equivalent electrical circuit with resistances, capacitors and a constant phase element (CPE), shown in Fig. 10a,b. The equivalent impedances are given by Eqs. (1) and (2) for HYD-T and CAN-T materials, respectively:

1 $$Z=\frac{1}{\frac{1}{{Z}_{C1}}+\frac{1}{{R}_{3}}}+\frac{1}{\frac{1}{{Z}_{C2}}+\frac{1}{\frac{1}{\frac{1}{{R}_{1}}+\frac{1}{{R}_{2}}}+{Z}_{CPE}}}$$

2 $$Z={Z}_{C1}+{R}_{3}+\frac{1}{\frac{1}{{Z}_{C2}}+\frac{1}{{Z}_{C3}}+\frac{1}{\frac{1}{\frac{1}{{R}_{1}}+\frac{1}{{R}_{2}}}+{Z}_{CPE}}}$$

where *Z*_*C*_ = *1/jωC*, Z_CPE_ = 1/P(j(*ω − ω*’))^α^. The CPE element is explained in terms of a fractional parameter *α* and a parameter *P* = *ηC*, in which *η *is a calibration function, where *R*, *C*, *P,* and *α* are adjustable parameters. Α takes a value of + 1 or − 1 for a capacitive or inductive reactive impedance, respectively, and determines the meaning of *P*^36^.

The adjustable parameters are reported in Tables 2 and 3 for HYD-T and CAN-T samples, respectively. Fitted parameters between light and dark conditions exhibit differences expressed as percentages, registered in Table 2. The difference was calculated according to the equation^36^:

$$ \Delta \, = \,\left( {{\text{Parameter in darkness}} - {\text{ Parameter under illumination}}} \right)/ \, ({\text{Parameter in darkness}}). $$

**Table 2:** Values of the parameters of the electric circuit model obtained from the fitted Nyquist plots for HYD-T materials in darkness, under illumination, and the difference between them.

Sample	C1 (pF)	C2 (μF)	R1 (Ω)	R2 (Ω)	R3 (kΩ)	P	α
In dark							
HYD-T-8.7	54.32	7.17	45,133	1.6 × 10^–12^	1.5	1.38 × 10^–5^	0.42
HYD-T-25	55.86	7.38	9 × 10^–07^	8.5 × 10^–11^	2.9	1.19 × 10^–19^	0.05
HYD-T-33.25	58.66	5.97	2 × 10^–11^	2517.2	4.0	4.83 × 10^–6^	0.67
HYD-T-41.3	57.80	3.75	1 × 10^–15^	1.0 × 10^5^	2.9	1.03 × 10^–5^	0.74
HYD-T-49.45	57.29	6.77	4 × 10^–22^	1.2 × 10^–10^	4.4	1.36 × 10^–6^	0.61
Under illumination							
HYD-T-8.7	44.70	4.71	1 × 10^–13^	1 × 10^–12^	1.9	9.68 × 10^–6^	0.62
HYD-T-25	48.30	5.27	1 × 10^–11^	2 × 10^–11^	3.6	7.61 × 10^–6^	0.51
HYD-T-33.25	68.79	0.33 × 10^–3^	6915	817	3.5	1.46 × 10^–5^	0.83
HYD-T-41.3	54.08	2.74	704	2246	3.2	1.02 × 10^–5^	0.79
HYD-T-49.45	54.77	0.54	6 × 10^–4^	2 × 10^–13^	4.8	1.31 × 10^–5^	0.84

Difference							
Sample	ΔC1 (%)	ΔC2 (%)	ΔR1 (%)	ΔR2 (%)	ΔR3 (%)	ΔP (%)	Δα (%)
HYD-T-8.7	18	34	100	38	−27	30	−48
HYD-T-25	14	29	100	76	−24	−6 × 10^15^	−920
HYD-T-33.25	−17	100	−3 × 10^16^	68	13	−202	−24
HYD-T-41.3	6	27	−7 × 10^15^	−2 × 10^10^	−10	1	−7
HYD-T-49.45	4	92	−2 × 10^20^	100	−9	−863	−38

**Table 3:** Values of the parameters of the electric circuit model obtained from the fitted Nyquist plots for CAN-T materials in darkness, under illumination, and the difference between them.

Sample	C1 (μF)	C2 (pF)	C3 (pF)	R1 (kΩ)	R2 (kΩ)	R3 (Ω)	P	α
In dark								
CAN-T-8.7	21.73	35.41	24.61	2.3	1.2	1.2 × 10^–12^	5.19 × 10^–5^	0.75
CAN-T-25	493.74	16.12	47.61	1.7	5.1	1.9 × 10^–14^	1.36 × 10^–5^	0.91
CAN-T-33.25	130.29	41.95	19.37	2.2	4.1	2.0 × 10^–25^	1.34 × 10^–5^	0.91
CAN-T-41.3	500.00	45.64	19.36	4.6	2.7	2.1 × 10^–24^	1.33 × 10^–5^	0.92
CAN-T-49.45	499.85	13.24	45.35	4.5	9.8	1.5 × 10^–13^	9.11 × 10^–6^	0.96
Under illumination								
CAN-T-8.7	16.36	47.54	11.49	4.4	1.1	5.1 × 10^–13^	7.1 × 10^–5^	0.69
CAN-T-25	49.52	49.57	12.74	2.4	3.3	7.8 × 10^–13^	2.2 × 10^–5^	0.83
CAN-T-33.25	36.29	48.71	8.69	3.0	3.5	3.0 × 10^–25^	2.1 × 10^–5^	0.82
CAN-T-41.3	61.24	61.53	1.08	5.0	3.0	3.1 × 10^–23^	2.0 × 10^–5^	0.84
CAN-T-49.45	16.30	41.09	15.77	9.1	5.1	8.1 × 10^–13^	3.9 × 10^–5^	0.75

Difference								
Sample	ΔC1 (%)	ΔC2 (%)	ΔC3 (%)	ΔR1 (%)	ΔR2 (%)	ΔR3 (%)	ΔP (%)	Δα (%)
CAN-T-8.7	25	−34	53	−91	8	58	−37	8
CAN-T-25	90	−208	73	−41	35	−4005	−62	9
CAN-T-33.25	72	−16	55	−36	15	−50	−57	10
CAN-T-41.3	88	−35	94	−9	−11	−1376	−50	9
CAN-T-49.45	97	−210	65	−102	48	−440	−328	22

It is important to elucidate the electrical conduction mechanism for TiO_2_@zeolite materials; however, it could be challenging to solve the exact formation mechanism. This study investigated this issue considering the following facts: (a) The distortion of the semicircles is attributed to the contribution of grain and grain boundaries. (b) A larger curvature radii in the Nyquist plots suggest a lower charge transfer at the electrolyte/electrode interface^37–41^. The radius of the semicircles increases when the TiO_2_ amount increases in both HYD-T and CAN-T samples, which indicates that the resistance of the entire system increases; thus, there is an increase in the charge transfer resistance crosswise the solid–liquid interface. Those resistances increase when the TiO_2_ amount increases and the resistance increases in each sample when the materials are illuminated. (c) The increase in the system resistance when the quantity of TiO_2_ increases indicates that the agglomeration of TiO_2_ particles (as depicted in SEM results) increases the resistance between the TiO_2_-substrate interface. In addition, the increases in the agglomeration of TiO_2_ particles increase the resistance in the TiO_2_. (d) The increase in the resistance of the system when illuminated indicates that the generated photoelectrons into TiO_2_ increase the charge concentration in the surface; it increases the capacitance contribution to the impedance of the system. Additionally, the radicals originated in the solution by oxidation processes are adsorbed by the substrate, or the generated photoelectrons on TiO_2_ recombine in the TiO_2_@zeolite solid.

According to the previous facts it is suggesting the following coherent mechanism:

For both series of samples, HYD-T and CAN-T, the value of ΔR2 is mostly positive (except for HYD-T-41.3) and it tends to increase when the TiO_2_ amount increases in the material, which means that resistance under illumination decreases, in comparison with resistance in darkness. This resistance is attributed to the resistance of the TiO_2_ because the density of electrons in the conduction band increases by the promotion of electrons from the valence band by light absorption. The value of ΔR2 is larger in the HYD-T than in the CAN-T samples, which suggests that there is a larger density of electrons on the surface of TiO_2_ in HYD-T than in the CAN-T samples. With this in mind, the capacitance associated with the surface of the TiO_2_ must increase when the material is illuminated; thus, the value of ΔC must be negative. In the CAN-T samples, ΔC2 behaves as expected; however, in HYD-T samples, the values of ΔC1 and ΔC2 are positive. These results suggest a larger electron transfer from the hybrid material to the solution in the HYD-T samples than in the CAN-T ones. Furthermore, the equivalent circuit in the HYD-T samples is two capacitors instead of the three capacitors in the CAN-T ones. Thus, the TiO_2_ -substrate in HYD-T fosters the charge transfer and avoids the charge accumulation in the interface TiO_2_ /substrate and subsequently in the interface hybrid-material/solution (see Fig. 10c). Conversely, in CAN-T samples, there is a charge accumulation in the interface TiO_2_ /substrate that avoids the charge transfer from the hybrid material to electrolyte and exhibits larger electron–hole recombination in the TiO_2_, especially in samples with wt% TiO_2_ larger than 25%, in which the agglomeration of TiO_2_ particles (SEM Figs. 5 and 6) fosters electron–hole recombination. Thus, the C2 is the capacitance in the TiO_2_/substrate interface in CAN-T samples. C1 is the capacitance in the electrode/solution, and R3 is the resistance in the solution; ΔC1 and ΔR3 exhibit positive values, indicating that there is a decrease of the charge accumulation in this interface and a decreasing of charge carriers in the solution due to charge transfer from TiO_2_ and electrolyte towards the substrate to neutralize the active sites in the zeolite phases. Thus, R1 is associated with the resistance of the substrate, and C3 is the capacitance in TiO_2_/substrate interface in CAN-T hybrid materials (see Fig. 10d). The decrease in the values of C2 under illumination (i. e., the decreasing of charge accumulation between sample/electrolyte and due to the charge transfer to promote oxidation and reduction reactions in the TiO_2_ surface to generate free radicals and ˙OH, the larger quantity of free radicals) is generated by HYD-T-8.7 and HYD-T-25. These samples exhibited the largest dye degradation among all samples^30^.

## Conclusions

This work studied the surface and electrical properties of the previously synthesized TiO_2_@zeolite materials used to degrade methyl orange, in which the zeolite was obtained from CFA. XPS results verified that TiO_2_ corresponds to anatase with the presence of the Ti^3+^ state. The morphological characteristics studied by SEM images show that the TiO_2_ particles agglomerate, forming spherical particles. The electric behavior studied by ALIS measurements allows us to conclude that the resistance of the entire system increases when the amount of TiO_2_ increases. According to the XPS, SEM and ALIS results the electrical behavior mainly depends on the interactions between substrate- TiO_2_ and substrate/ TiO_2_-liquid rather than the presence of Ti^3+^ in the surface. The best TiO_2_-substrate attachment in the CAN-T samples increases the capacitive behavior between the substrate and TiO_2_ interface; however, the resistance of the entire system is smaller than in HYD-T samples. Conversely, the low degree of TiO_2_-substrate attachment in the HYD-T samples decreases the capacitive behavior between the substrate and TiO_2_-interface, allowing a higher transfer of the charges between the solid–liquid interface. From the results a coherent electrical mechanism to explain the differences in the methyl orange degradation by both HYD-T and CAN-T samples was proposed, finally, the electric behavior of the hybrid materials produced from CFA and TiO_2_ is a complex mechanism that requires additional studies to be completely elucidated.

## Data Availability

All data generated or analyzed during this study are included in this published article.
